# High Maternal Omega-3 Supplementation Dysregulates Body Weight and Leptin in Newborn Male and Female Rats: Implications for Hypothalamic Developmental Programming

**DOI:** 10.3390/nu13010089

**Published:** 2020-12-30

**Authors:** Soniya Xavier, Jasmine Gili, Peter McGowan, Simin Younesi, Paul F. A. Wright, David W. Walker, Sarah J. Spencer, Luba Sominsky

**Affiliations:** 1 School of Health and Biomedical Sciences, RMIT University, Melbourne, VIC 3083, Australia; soniya.xavier@student.rmit.edu.au (S.X.); jasminegili97@hotmail.com (J.G.); Simin.Younesi@student.rmit.edu.au (S.Y.); paul.wright@rmit.edu.au (P.F.A.W.); David.Walker@rmit.edu.au (D.W.W.); Luba.Sominsky@rmit.edu.au (L.S.); 2 School of Science, RMIT University, Melbourne, VIC 3001, Australia; peter.mcgowan@rmit.edu.au; 3 ARC Centre of Excellence for Nanoscale Biophotonics, RMIT University, Melbourne, VIC 3001, Australia

**Keywords:** omega-3, pregnancy, development, leptin, hypothalamus

## Abstract

Maternal diet is critical for offspring development and long-term health. Here we investigated the effects of a poor maternal diet pre-conception and during pregnancy on metabolic outcomes and the developing hypothalamus in male and female offspring at birth. We hypothesised that offspring born to dams fed a diet high in fat and sugar (HFSD) peri-pregnancy will have disrupted metabolic outcomes. We also determined if these HFSD-related effects could be reversed by a shift to a healthier diet post-conception, in particular to a diet high in omega-3 polyunsaturated fatty acids (ω3 PUFAs), since ω3 PUFAs are considered essential for normal neurodevelopment. Unexpectedly, our data show that there are minimal negative effects of maternal HFSD on newborn pups. On the other hand, consumption of an ω3-replete diet during pregnancy altered several developmental parameters. As such, pups born to high-ω3-fed dams weighed less for their length, had reduced circulating leptin, and also displayed sex-specific disruption in the expression of hypothalamic neuropeptides. Collectively, our study shows that maternal intake of a diet rich in ω3 PUFAs during pregnancy may be detrimental for some metabolic developmental outcomes in the offspring. These data indicate the importance of a balanced dietary intake in pregnancy and highlight the need for further research into the impact of maternal ω3 intake on offspring development and long-term health.

## 1. Introduction

Pre-conception and pregnancy diets are important for offspring development and long-term health [1]. It is now widely understood that a Western-style diet high in fat and sugar before and after conception can lead to accelerated weight gain, obesity, and an increased prevalence of type 2 diabetes in the offspring [2,3,4]. For example, increased consumption of processed foods high in fat and sugar during pregnancy is strongly associated with overweight and obesity in children [5]. While the lifelong dietary patterns of children are likely to be influenced by parental food choices [6], rodent studies confirm that the influences of maternal peri-pregnancy diet can be independent of either maternal obesity or offspring post-weaning diet and can be retained in the long term. As such, a maternal diet high in fat and sugar compromises feto-placental growth and glucose tolerance in mice [7], leading to increased body weight, insulin resistance and other symptoms of metabolic dysfunction later in life [8,9,10].

The effects of maternal diet on offspring metabolic development and function appear to be dependent upon both the pre- and post-conception stages and the sex of the conceptus [11]. Exposure to a high-fat diet before conception alone alters offspring body weight and fat distribution in both males and females, whereas maternal overnutrition pre-conception and throughout pregnancy increases the incidence of metabolic dysfunction and metabolic inflammation in male, but not female offspring [11]. It is likely that sex differences in placental development, the epigenome and the hormonal environment of pregnancy contribute to this sexual dimorphism in metabolic malprogramming [12].

In addition to peripheral mechanisms including excess fat deposition, insulin resistance and pancreatic β cell dysfunction [8], offspring obesity and comorbidities are likely to be due to changes in the developing brain driven by peri-pregnancy maternal diet, including disrupted programming of hypothalamic satiety signalling [13]. The establishment of hypothalamic satiety-regulatory circuitry occurs during the first two weeks of life in rodents [14], driven by the trophic actions of the leptin surge on the hypothalamus between postnatal day (P) 4 and 14 [15,16]. A maternal diet high in fat and sugar can lead to an increased and prolonged leptin surge, resulting in aberrant maturation of satiety signalling networks, leptin resistance, altered hypothalamic function, and overweight later in life [17]. Although the hypothalamic satiety-regulatory circuitry is functionally immature at birth in the rat, most hypothalamic neurons have already migrated to their adult destination, where they express the appropriate neurotransmitters and neuropeptides [13]. Maternal overnutrition has been shown to alter circulating leptin levels and gene expression of key hypothalamic appetite regulators, such as neuropeptide Y (NPY) and pro-opiomelanocortin (POMC), on P1, one day after birth [18], suggesting that the influences of maternal diet on the establishment of hypothalamic satiety signalling take place during intrauterine development. On the other hand, the postnatal brain can be remarkably resilient and adaptable to perinatal insults. For instance, supplementation with leptin during the early postnatal period re-establishes hypothalamic connectivity in leptin deficient *ob/ob* mice [15], and reverses the obese phenotype in the offspring of undernourished rats [19]. Consumption of a healthy diet postweaning, following a period of postnatal overnutrition, also reverses neonatal leptin resistance and aberrant hypothalamic neuronal signalling in the adult brain [20,21]. However, the extent to which the negative effects of a poor maternal diet prior to and during pregnancy on the developing brain of the offspring can be reversed by improving the post-conception diet is not yet clear.

Here, we determined the proximal effects of a poor maternal diet during the pre-conception period on male and female offspring health and potential capacity for satiety signalling immediately after birth, and if any dysfunction could be reversed by reverting to a putatively healthy diet post-conception. We also determined if maternal consumption of a diet high in omega-3 polyunsaturated fatty acids (ω3 PUFAs), known to be essential for the developing brain and to have a critical role in cognitive, neuropsychiatric and metabolic development [22,23], resulted in benefits for the offspring at birth. During foetal and neonatal life, there is a limited capacity for PUFAs synthesis, and ω3 PUFAs are supplied via the maternal intake [24]. Maternal consumption of a diet rich in ω3 PUFAs reduces body fat mass and insulin resistance in adult rat offspring [25,26]. Dietary supplementation with ω3 PUFAs during pregnancy and lactation has also been shown to protect from neurodegeneration and memory impairments in adult rat offspring [27], and to improve infant attention [28] and problem-solving [29] in humans. The effects of maternal ω3 PUFAs on the development of hypothalamic satiety signalling remain unexplored. Here, we determined the effects of an increased intake of ω3 PUFAs post-conception on the developing hypothalamus in male and female offspring in the context of a control (chow) or high-fat-high-sugar diet (HFSD) pre-conception. We hypothesised that maternal peri-pregnancy HFSD consumption would lead to poor metabolic outcomes in the offspring at birth, and that these would be reversed by ω3 dietary intake during pregnancy. This study design attempted to mimic a human setting, where, upon confirmation of pregnancy, women consuming a poor diet would be likely to either pursue their existing poor eating habits or to make a pregnancy-inspired choice to engage in a healthier lifestyle.

## 2. Methods

### 2.1. Animals

All experiments were conducted in accordance with the Australian Code of Practice for the Care and Use of Animals for Scientific Purposes, with approval from the RMIT University Animal Ethics Committee (AEC1808). In these experiments, 53 sexually mature (6 weeks of age) young female Wistar rats were obtained from the Animal Resources Centre, Perth, WA, Australia. On arriving at the RMIT University Animal Facility, they were housed at 22 °C on a 12 h light cycle (7 a.m. to 7 p.m.) and acclimatised for approximately one week prior to the experiment. During this time, we provided them with *ad libitum* pelleted rat chow and water. After acclimatization, female rats were assigned to receive either their control chow diet (4.8% fat, Meat Free Rat and Mouse Diet, #12111, Specialty Feeds, Glen Forrest, WA, Australia) or a diet high in saturated fat and carbohydrate (HFSD: 20% fat derived from lard, #SF04-025, Specialty Feeds) for 8 weeks. See Table 1 for complete composition of diets.

### 2.2. Mating and Pregnancy Dietary Protocol

**Figure 1:**
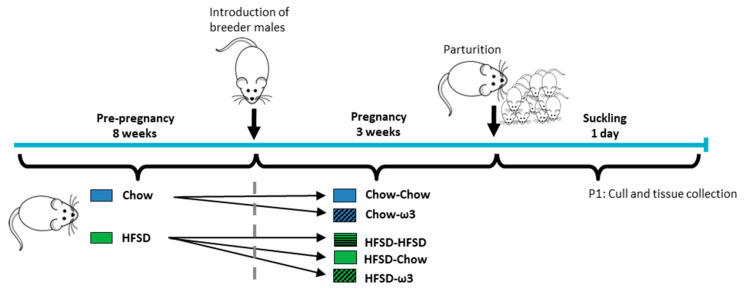
Experimental timeline. Six-week-old female rats were given either chow or high-fat-high-sugar diet (HFSD) for 8 weeks before the introduction of adult male studs. Following mating, female rats were separated into the following dietary regimens: those fed chow pre-conception were allocated to receive either chow or ω3-enriched diets. Those fed HFSD pre-conception were allocated to either continue HFSD consumption during pregnancy, or to receive chow or ω3 diets, resulting in the following group allocations: chow-chow; chow-ω3; HFSD-HFSD; HFSD-chow; HFSD-ω3. On postnatal day 1 (P1), dams and pups were culled and tissue collected for analyses.

After 8 weeks’ consumption of their respective diets, the females (6–10 per group) were harem-mated (3–4 same-diet females per male; a total of 12 adult male Wistar rats, at approximately 10 weeks of age) for three consecutive days. Following mating, the females were separated into cages of 2–3 and into the following dietary regimens: those fed chow preconception were allocated to receive either a chow or an ω-3-enriched diet (5% fat, high ω3, #SF09-109, Specialty Feeds; Table 1). Those fed HFSD preconception were allocated to either chow, HFSD, or ω3, resulting in the following pre/post-conception group allocations: chow-chow; chow-ω3; HFSD-chow; HFSD-HFSD; HFSD-ω3. We designed the study to mimic a human setting where women who consume a healthy and balanced diet prior to conception are unlikely to worsen their dietary habits upon conception to consume a poor diet during pregnancy. We therefore deliberately excluded the chow-HFSD group from the study design. For a detailed timeline of the experimental design, see Figure 1.

### 2.3. Tissue Processing

On P1 (one day after birth), litter size, male and female ratio, neonatal weights and nasal-anal length measurements were recorded. Individual rat weights and nasal-anal lengths were used to calculate a rat body mass index (rBMI) using the formula weight (g)/length (cm)^2^, as previously described [30]. All pups were culled by rapid decapitation for tissue collection. Trunk blood was collected and pooled with that of the other same-sex littermates for the assessment of metabolic hormones. Fixed brains were collected from one pup per sex per litter. Brains were immersion-fixed overnight in 4% paraformaldehyde in phosphate-buffered saline (PBS; pH 7.4), followed by washes in 70% ethanol prior to being stored in the same solution until processing. Snap-frozen brains were also collected from one pup per sex per litter, hypothalamus was dissected for gene expression analysis and the remainder of the brain used for fatty acid composition assessment. Dams and excess pups were also culled and used for other studies.

### 2.4. Measurement of Fatty Acid Composition

The method for assessing fatty acid composition was adapted from Jones et al. [31], with some modifications. Whole frozen brains (without the hypothalamus, see above) from one pup per sex from 3–4 litters per treatment were weighed and approximately 200 mg of tissue was ground to fine powder with a mortar and pestle using liquid nitrogen to maintain a frozen sample mass. Tissue samples were extracted using 10 mL of dichloromethane (DCM): methanol (2:1 vol:vol). Residual water in sample extract volumes was removed by addition of 1 g ± 0.1 g of anhydrous Na_2_SO_4_ with gentle agitation. Total lipid extracts were methylated with the addition of 2 mL of 4% H_2_SO_4_ in mass spectrometry grade methanol, mixed by agitation and heat at 50 °C for 45 min. Extracts were filtered through a microcolumn with DCM-rinsed glass wool and a bed of 1.0 ± 0.1 g of anhydrous Na_2_SO_4_. Eluate was collected and evaporated to 0.5 mL under a stream of N_2_ using a Zymark TurboVap LV Evaporator with a water bath at 60 °C. The sample was then diluted to 2 mL with DCM and the lower DCM layer aliquoted into a gas chromatography (GC) vial. Fatty acid methyl esters (FAMEs) analysis was performed using Agilent Technologies model 6890 GC with flame-ionization detection. The column was a 100 m × 0.25 mm × 0.25 μm Supelco SP-2560 (Bellefonte, PA, USA) with the following temperature program: 100 °C (4 min), to 200 °C at a rate of 25 °C/min (held for 8 min), to 250 °C at 5 °C/min (held for 6 min). Hydrogen was used as the carrier gas with an inlet split ratio of 5:1. Target analyte quantitation was performed referencing an external standard response for a seven-point linear calibration for Certified Reference Material—Supelco 37 Component FAME Mix (CRM47885).

### 2.5. Circulating Hormones

We assessed serum leptin concentrations using the Milliplex^®^ MAP Rat Cytokine/Chemokine Magnetic Bead Panel assay (Merck Millipore, Billerica, MA, USA). The data were extracted using Bio-Plex Manager software and analysed in Prism (Graphpad Software Inc., San Diego, CA, USA), as per the manufacturer’s instructions. The lowest limit of detection was 10.2 pg/mL, and the intra-assay and inter-assay coefficients of variation (CVs) were 3.4% and 14.3%, respectively.

To assess circulating triglycerides, we performed a triglyceride colorimetric assay (Cayman Chemical, Ann Arbor, MI, USA), following the manufacturer’s instructions. Intra-assay variability was 1.34% CV and lower limit of detection was 0.5 mg/dL.

To determine serum ghrelin concentrations, we performed a standard Rat/Mouse Ghrelin (total) ELISA (Merck Millipore), following the manufacturer’s instructions. The lowest limit of detection was 0.04 ng/mL and the intra-assay variability for total serum ghrelin was 0.7–1.3% CV. We were unable to assess acyl ghrelin levels in this study, due to the limited sample availability and thus inability to inhibit protease activity and acidify the sample [32].

We also measured circulating growth hormone levels, using a Rat/Mouse Growth Hormone ELISA (Merck Millipore). The intra-assay variability was 1.7–4.3% CV, and the lowest limit of detection was 0.07 ng/mL. All samples were processed in the same plate for all assays.

To complement our metabolic findings in the pups, we also assessed circulating metabolic hormones from samples collected from the dams. On P1, we deeply anesthetized the dams without recovery with sodium pentobarbital (Lethabarb, Virbac Australia; ~150 mg/kg, i.p.), and collected cardiac blood. Blood samples were placed in EDTA-coated tubes over ice, and after centrifugation, the plasma supernatant was stored at −20 °C until analysis. Leptin and triglyceride concentrations were assessed as described above. We assayed insulin in EDTA plasma samples using a standard insulin ELISA (Merck Millipore), following the manufacturer’s instructions; intra-assay variability was 0.9–8.4% CV. To assess circulating total and acyl ghrelin, cardiac blood was placed directly into a centrifuge tube with no anti-coagulant and treated with Pefabloc SC (protease inhibitor, 1 mg/mL; Roche Diagnostics, Manheim, Germany). We then left the blood to clot for 30 min, centrifuged the samples at 2500× *g* for 15 min at 4 °C, transferred the serum into a new tube and acidified it with HCl to a final concentration of 0.05 M. We performed standard ghrelin ELISAs for total and acyl ghrelin (Merck Millipore), following the manufacturer’s instructions. Intra-assay variability for acyl ghrelin was 0.3–7.0% CV, and the lower limit of detection was 0.008 ng/mL. We subtracted acyl ghrelin concentrations from total ghrelin concentrations to obtain a value for des-acyl ghrelin [33].

### 2.6. NPY Immunohistochemistry

Fixed brains from the pups were paraffin-embedded and sectioned into 10 μm sections. Histology services were provided by the University of Melbourne’s histology facility (Parkville, VIC, Australia). We de-paraffinized the brain sections in histolene (3 × 5 min) and rehydrated them in decreasing concentrations of ethanol. We performed an antigen retrieval in sodium citrate buffer (10 mM sodium citrate, pH = 6), where sections were then microwaved at full power (800 W), for approximately 3 min at 85–90 °C. Sections were then blocked in 3% bovine serum albumin (BSA; 0.3% Triton X-100/PBS) for 1 h at room temperature, followed by application of primary NPY antibody (1:500, rabbit-monoclonal; Sigma-Aldrich, St. Louis, MO, USA) for 18 h. The sections were then washed and incubated with the secondary antibody (Alexa-Fluor 594 goat-anti-rabbit; 1:500, Thermo Scientific, Rockford, IL, USA) for 2 h at room temperature in the dark. We then coverslipped the slides using Fluoroshield with DAPI mounting medium (Sigma-Aldrich) and stored the slides at 4 °C until imaging.

### 2.7. Immunohistochemistry Analysis

Photomicrographs of the sections of interest were taken using an Olympus upright microscope (Olympus BX61 fluorescent microscope) with a 20× objective lens fitted with a Nikon DS-Ri camera and LabSense image capture software v1.6 (Olympus, Tokyo, Japan). Images were taken at 1608 × 1608-pixel density, and the image file imported into ImageJ analysis software (National Institute of Health (NIH), Bethesda, MD, USA) to be processed. Sections were assessed by an experimenter who was blinded to the treatment groups. We assessed the density of NPY fibres using a thresholding method as previously described [21,34]. Briefly, lower and upper threshold limits were detected on brain sections from the control group animals and settings were then kept constant to the mean of the control thresholds throughout the (blinded) analysis. For the arcuate nucleus of the hypothalamus (ARC) we assessed three sections, approximately 252 μm apart, between 2.04 to 2.16 mm caudal to the bregma for each animal. For the paraventricular nucleus of the hypothalamus (PVN), we assessed three sections between 1.80 and 2.04 mm caudal to the bregma. Data are presented as the mean of these three values.

### 2.8. Gene Expression

We assessed changes in gene expression as previously described [35,36,37,38]. Briefly, we extracted RNA from the hypothalamus using QIAzol reagents and RNeasy Mini Kit (Qiagen, Valencia, CA, USA). RNA concentrations and quality were assessed using a spectrophotometer (NanoDrop 2000/2000c; Thermo Fisher Scientific, Wilmington, DE, USA). cDNA was synthesised using the QuantiTect Reverse Transcription kits (Qiagen) as per the manufacturer’s instructions. qRT-PCR was then performed using Taqman Gene Expression Assays (Thermo Fisher Scientific) on a QuantStudio 7 Flex instrument (Applied Biosystems). We assessed changes in the expression of the leptin receptor (*Lepr*), *Npy*, *Agrp*, *Pomc* and growth-hormone secretagogue receptor (*Ghsr*). Primer details are shown in Table 2. We compared a relative quantitative measure of the target gene expression with the expression of an endogenous control, *Gapdh*. mRNA expression was analysed using the equation 2^−ΔΔ*C*(*t*)^, where *C*(*t*) is the threshold cycle at which fluorescence is first detected significantly above background [39].

### 2.9. Statistical Analyses

Statistical analyses were conducted using Statistical Package for the Social Sciences for Windows (SPSS Inc., Chicago, IL, USA). Graphs were generated using GraphPad Prism software (GraphPad software, Inc., San Diego, CA, USA). All data were analysed using two-way analyses of variance (ANOVAs), with pre-pregnancy and pregnancy diets as independent variables. Where significant interactions or significant main effects of pregnancy diets were found, Tukey’s HSD *post hoc* tests were performed. We also conducted Pearson’s correlation analysis between the levels of circulating leptin and body weight. We analysed male and female data separately, due to the known sex differences in hypothalamic development [40]. Data are presented as the mean ± SEM. Statistical significance was assumed when *p* < 0.05.

## 3. Results

### 3.1. Effects of Maternal Pre- and Post-Conception Diet on Pup Survival and Morphometric Outcomes

**Figure 2:**
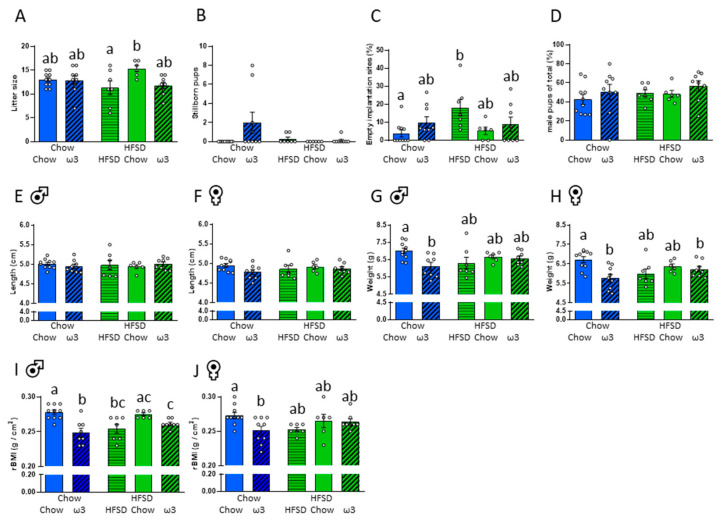
Effects of maternal pre- and post-conception diet on pup survival and morphometric outcomes. (**A**) Total litter size. (**B**) Number of stillborn pups. (**C**) Percentage of implantation sites additional to the number of pups born. (**D**) Sex ratio (% of male pups). (**E**) Pup length, males. (**F**) Pup length, females. (**G**) Pup weight, males. (**H**) Pup weight females. (**I**) Rat body mass index (rBMI), males. (**J**) rBMI, females. HFSD: high-fat-high-sugar diet. Letters denote *post hoc* differences between groups (*p* < 0.05). Data are mean ± SEM (*n* = 6–10). Panels A–D: Each data point represents the mean of one litter. Panels E–J: Each data point represents the mean of one litter per sex.

As anticipated, consumption of HFSD for 8 weeks significantly increased maternal body weight pre-conception (chow = 117.8 ± 4.3 g, HFSD = 141.4 ± 4.2 g; t_(51)_ = 3.95, *p* < 0.001). Consumption of HFSD diet only during the pre-conception phase did not affect pregnancy outcomes or pup morphometrics (Figure 2A–D). That is, there were no effects of pre-conception diet on litter sizes, mortality, the ratio of foetal implantation sites, sex ratios of live pups, pup weights, lengths, or rat body mass indices.

Maternal diet during pregnancy, however, had some notable effects on offspring morphometrics. Litter sizes were smaller from those dams that had HFSD both pre-conception and during pregnancy relative to those that were switched to chow after conception (significant pre- and post-conception diet interaction F_(1,35)_ = 4.01, *p* = 0.053; Figure 2A). There were no significant differences between the groups in the number of stillborn pups (Figure 2B). However, HFSD both pre-conception and continued during pregnancy significantly increased the incidence of implantation sites that were additional to the number of pups born, relative to those seen in the dams fed chow throughout, indicating that there were additional conceptuses that did not develop through to parturition in this group (significant effect of post-conception diet: F_(2,35)_ = 4.23, *p* = 0.023; Figure 2C). The sex ratio of the pups was not different between the groups (Figure 2D).

Pup lengths at P1 were not influenced by diet pre- or post-conception (Figure 2E,F), but there was a significant effect on weight. Thus, ω3 consumed during pregnancy reduced both male and female pup weight in those that had normal chow prior to pregnancy, relative to those fed chow throughout. In females, but not in males, ω3 consumption also reduced body weight in those that had HFSD prior to pregnancy relative to chow-only-fed controls (males: significant effect of post-conception diet: F_(2,34)_ = 3.32, *p* = 0.048; females: significant effect of post-conception diet: F_(2,35)_ = 5.06, *p* = 0.012, significant pre- and post-conception diet interaction: F_(1,35)_ = 4.90, *p* = 0.033; Figure 2G,H).

Changes in offspring weight with maternal diet were reflected in the rBMI (males: significant effect of post-conception diet: F_(2,34)_ = 13.78, *p* < 0.001; females: significant effect of post-conception diet: F_(2,35)_ = 3.16, *p* = 0.055; Figure 2I,J). As such, ω3 consumed during pregnancy reduced both male and female pup rBMI in those that had chow prior to pregnancy relative to those maintained on chow throughout. In males, rBMI was similarly reduced in those that had HFSD pre-conception and either HFSD or ω3 post-conception relative to those fed normal chow throughout.

### 3.2. Effects of Maternal Pre- and Post-Conception Diet on Brain Fatty Acid Composition

**Figure 3:**
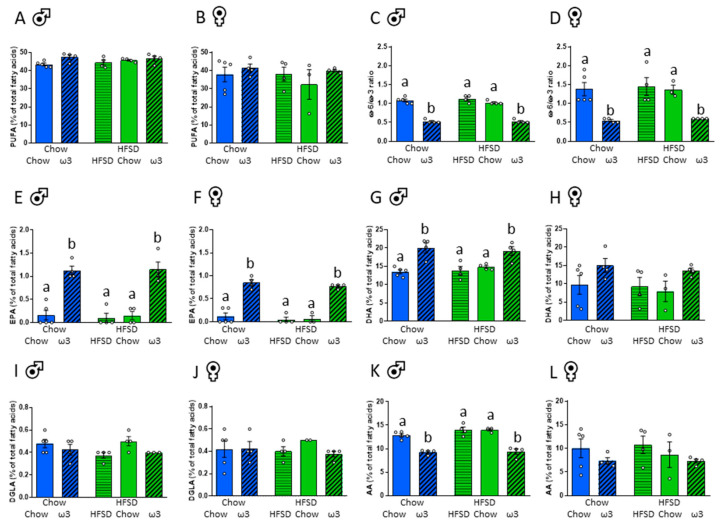
Effects of maternal pre- and post-conception diet on brain fatty acid composition. (**A**) Total polyunsaturated fatty acids (PUFAs), males. (**B**) Total PUFAs, females. (**C**) Ratio of ω6 to ω3 PUFAs, males. (**D**) Ratio of ω6 to ω3 PUFAs, females. (**E**) Eicosapentaenoic acid (EPA), males. (**F**) EPA, females. (**G**) Docosahexaenoic acid (DHA), males. (**H**) DHA, females. (**I**) Dihomogammalinolenic acid (DGLA), males. (**J**) DGLA, females. (**K**) Arachidonic acid (AA), males. (**L**) AA, females. HFSD: high-fat-high-sugar diet. Letters denote *post hoc* differences between groups (*p* < 0.05). Data are mean ± SEM (*n* = 3–5), presented as a percentage of total fatty acids. Each data point represents one pup per litter per sex.

To determine whether the changes in pups’ body weight were likely to be driven by the altered placental nutrient transfer and foetal nutrient supply, we assessed the brain composition of ω3 and ω6 PUFAs. Maternal pre- or post-conception diet had no effect on brain composition of total PUFAs (Figure 3A,B). The composition of total ω3 PUFA, however, was strongly influenced by post-conception diet (males: significant effect of post-conception diet: F_(2,16)_ = 27.36, *p* < 0.001; females: significant effect of post-conception diet: F_(2,15)_ = 4.20, *p* = 0.036). In males, maternal consumption of high ω3 significantly increased the content of ω3 PUFAs irrespective of their pre-pregnancy diet (Supplementary Materials Table S1). There were no significant differences between the groups with *post hoc* comparisons in female brains (Supplementary Materials Table S2). Similarly, in males, but not females, ω6 PUFA composition was significantly reduced in those born to ω3-fed dams (males: significant effect of post-conception diet: F_(2,16)_ = 60.36, *p* < 0.001; Supplementary Materials Tables S1 and S2). The ratios between ω6 and ω3 PUFAs were markedly affected by post-conception diet (males: significant effect of post-conception diet: F_(2,16)_ = 153.67, *p* < 0.001, females: significant effect of post-conception diet: F_(2,15)_ = 17.36, *p* < 0.001; Figure 3C,D), with the ω6/ω3 ratio being decreased in the brains of both male and female pups born to dams fed a ω3-enriched diet during pregnancy relative to the other groups.

We then assessed the composition of the most biologically relevant ω3 and ω6 PUFAs, essential for foetal development; namely, eicosapentaenoic acid (EPA; 20:5ω-3), docosahexaenoic acid (DHA; 22:6ω-3), dihomogammalinolenic acid (DGLA; 20:3ω-6), and arachidonic acid (AA; 20:4ω-6; Figure 3E–L) [41]. Maternal diet of either chow or HFSD prior to conception once again had no effect on brain composition of these PUFAs. In contrast, EPA content was significantly influenced by post-conception consumption of ω3 (males: significant effect of post-conception diet: F_(2,16)_ = 47.55, *p* < 0.001, females: significant effect of post-conception diet F_(2,15)_ = 83.34, *p* < 0.001; Figure 3E,F). Similar effects of ω3 dietary intake during pregnancy on brain content of DHA were evident in male, but not female pups (males: significant effect of post-conception diet: F_(2,16)_ = 19.66, *p* < 0.001; Figure 3G,H). Post-conception ω3 diet did not significantly affect DGLA content (Figure 3I,J). While there were no significant effects of maternal diet during pregnancy on AA PUFA content in the female brain, post-conception consumption of ω3 reduced the content of this ω6 fatty acid in males (males: significant effect of post-conception diet: F_(2,16)_ = 58.87, *p* < 0.001; Figure 3K,L). Detailed fatty acid profiles are shown in Supplementary Materials Tables S1 and S2.

### 3.3. Effects of Maternal Pre- and Post-Conception Diet on Pup Circulating Metabolic Factors

To determine if maternal diet affected circulating metabolic factors in the neonate and thus the potential to alter the development of the hypothalamic satiety circuitry, we next examined circulating leptin levels in the pups. Although stomach weight and milk content were not measured, all of the live pups except two (both from the same litter where the dam was fed chow pre-conception, then ω3 post-conception) were seen to have milk in their stomachs at P1.

**Figure 4:**
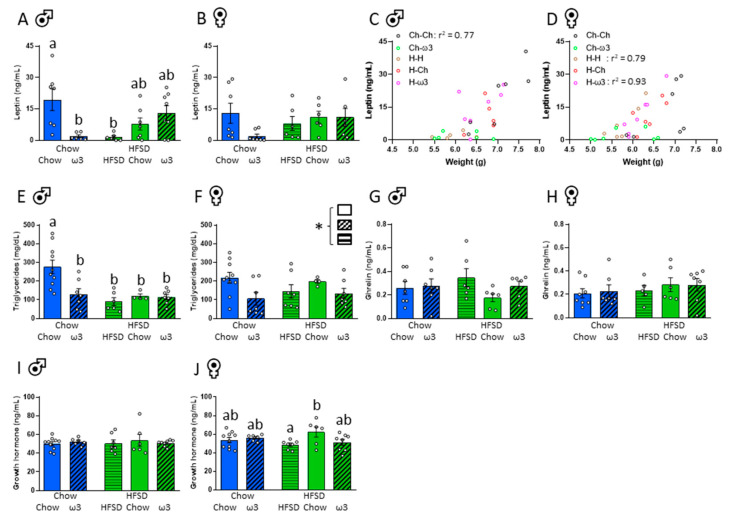
Effects of maternal pre- and post-conception diet on pup circulating leptin and ghrelin. (**A**) Circulating leptin, males. (**B**) Circulating leptin, females. (**C**) Correlation between circulating leptin and weight at postnatal day 1, males. (**D**) Correlation between circulating leptin and weight at postnatal day 1, females. (**E**) Circulating triglycerides, males. (**F**) Circulating triglycerides, females. (**G**) Circulating ghrelin, males. (**H**) Circulating ghrelin, females. (**I**) Circulating growth hormone, males. (**J**) Circulating growth hormone, females. HFSD: high-fat-high-sugar diet; Ch: chow (in (**C**,**D**)). Letters denote *post hoc* differences between groups; * significant effect of post-conception diet (*p* < 0.05). Data are mean ± SEM (*n* = 6–10). Each data point represents one pup per litter per sex.

Relative to chow-only-fed controls, circulating leptin was reduced in male pups (but not females) from dams fed HFSD pre-conception and during pregnancy, and in those fed a chow diet prior to conception and then an ω3-replete diet during pregnancy (significant pre- and post-conception diet interaction: F_(1,25)_ = 11.38, *p* = 0.002; Figure 4A,B). Circulating leptin was significantly correlated with pup weight in the chow-chow group in males (*r*^2^ = 0.77; *p* = 0.0094; Figure 4C) and the HFSD-HFSD (*r*^2^ = 0.79; *p* = 0.019), and HFSD-ω3 group in females (*r*^2^ = 0.93; *p* = 0.0005; Figure 4D). Triglycerides are important for neonatal growth [42], and in the male offspring, circulating triglycerides were suppressed in all groups, compared to chow-only-fed controls (significant pre- and post-conception diet interaction: F_(1,29)_ = 5.28, *p* = 0.029; Figure 4E). In females, triglyceride levels were also affected by post-conception diet (significant effect of post-conception diet: F_(2,30)_ = 3.44, *p* = 0.045; Figure 4F), with no *post hoc* differences.

At this early neonatal stage, circulating satiety hormone ghrelin levels were minimal as expected [43] and were not influenced by the pre- or post-conception maternal diet (Figure 4G,H). Growth hormone levels in males were also not affected by maternal diet (Figure 4I). In females, pups born to dams fed HFSD pre-conception, then chow post-conception had increased growth hormone levels relative to those fed HFSD from pre-conception and throughout the post-conception period (significant pre- and post-conception diet interaction: F_(1,32)_ = 4.40, *p* = 0.044; Figure 4J).

To determine whether changes in leptin and triglyceride levels seen in the pups corresponded to changes in these factors in maternal circulation, we also assessed their levels in maternal blood (Table 3). Unlike in the pups, HFSD pre-pregnancy led to an increase in circulating leptin levels in the dams, which was further increased by consumption of ω3 during pregnancy relative to rats fed chow during pregnancy (significant effect of pre-conception diet: F_(1,33)_ = 5.29, *p* = 0.028; significant effect of post-conception diet: F_(2,33)_ = 8.53, *p* = 0.001). Consumption of HFSD throughout the pre-conception and post-conception periods significantly increased maternal triglyceride levels and this increase was rescued by the switch to putatively healthier dietary normal chow or ω3 during pregnancy (significant effect of post-conception diet: F_(2,34)_ = 26.88, *p* < 0.001). There were no differences in insulin, or total or acyl ghrelin levels, while consumption of HFSD throughout the study led to an increase in des-acyl ghrelin relative to consumption of ω3 (significant effect of post-conception diet: F_(2,33)_ = 4.15, *p* = 0.024). See Table 3.

### 3.4. Effects of Maternal Pre- and Post-Conception Diet on the Hypothalamic Satiety System

**Figure 5:**
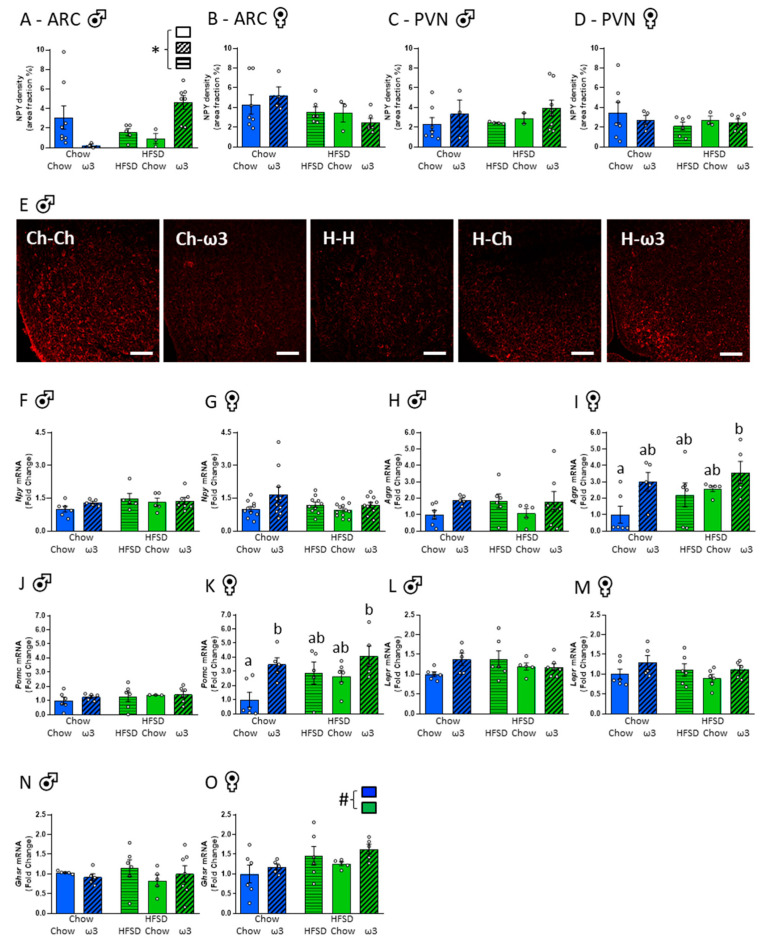
Effects of maternal pre- and post-conception diet on the hypothalamic satiety system. (**A**) Neuropeptide Y (NPY) density in the arcuate nucleus (ARC), males. (**B**) ARC NPY density, females. (**C**) Paraventricular nucleus (PVN) NPY density, males. (**D**) PVN NPY density, females. (**E**) Representative photomicrographs of NPY-positive cells in the ARC, males. Scale bar = 50 μm. (**F**) *Npy* mRNA, males. (**G**) *Npy* mRNA, females. (**H**) Agouti-related peptide (*Agrp)* mRNA, males. (**I**) *Agrp* mRNA, females. (**J**) Pro-opiomelanocortin (*Pomc*) mRNA, males. (**K**) *Pomc* mRNA, females. (**L**) Leptin receptor (*Lepr*) mRNA, males. (**M**) *Lepr* mRNA, females. (**N**) Growth hormone secretagogue receptor (*Ghsr*) mRNA, males. (**O**) *Ghsr* mRNA, females. HFSD: high-fat-high-sugar diet; Ch: chow (in **D**). Letters denote *post hoc* differences between groups; * significant effect of post-conception diet; ^#^ significant effect of pre-conception diet (*p* < 0.05). Data are mean ± SEM (*n* = 3–10). Each data point represents one pup per litter per sex.

Since our data suggested that the pups from a background of high ω3 in utero had restricted growth as indicated by reduced rBMI and, at least in males, this growth restriction was similar to that seen in the offspring of HFSD-fed dams, we next tested if maternal diet affected a key pathway in the development of hypothalamic satiety signalling. As expected [44,45], NPY fibres were sparse in the ARC at this early neonatal age. Pre-conception maternal diet did not significantly affect NPY density, but there was a significant effect of post-conception diet in males (F_(2,21)_ = 5.18, *p* = 0.015), with apparent opposing effects of ω3 in those fed chow versus HFSD pre-conception. However, there were no significant *post hoc* differences between the groups in males, and there were no significant effects of pre- or post-conception diets on ARC NPY density in females (Figure 5A,B,E). NPY fibres in the PVN were also sparse and there were no differences between the groups in this region (Figure 5C,D).

We also assessed hypothalamic expression of key metabolic neuropeptide and hormone receptor genes in the neonatal brain. There were no effects of maternal diet on the expression of *Npy*, agouti-related peptide (*Agrp*), proopiomelanocortin (*Pomc*), leptin receptor (*Lepr*) and the growth hormone secretagogue receptor (*Ghsr*) genes in the hypothalamus of male pups (Figure 5F,H,J,L,N). In females, there were similarly no differences in *Npy* and *Lepr* gene expression (Figure 5G,M). However, exposure to an ω3 diet post-conception after a HFSD consumption prior to pregnancy increased the expression of *Agrp* mRNA relative to those fed chow throughout (significant effect of post-conception diet: F_(2,22)_ = 4.04, *p* = 0.032; Figure 5I). The expression of *Pomc* mRNA was also increased in female pups born to ω3-fed dams relative to those fed chow throughout (significant effect of post-conception diet: F_(2,22)_ = 5.85, *p* = 0.009; Figure 5K). *Ghsr* was increased in females from dams fed a HFSD prior to conception (significant effect of pre-conception diet: F_(1,23)_ = 5.35, *p* = 0.030; Figure 5O). Collectively, these data suggest some sex differences in the effects of maternal peri-pregnancy diet on the developing metabolic signalling in the offspring and some detrimental effects of excess maternal ω3 intake on these developmental outcomes.

## 4. Discussion

In this study, we have shown novel evidence that consumption of an ω3-replete diet during pregnancy may alter several developmental parameters. As such, pups born to ω3-fed dams weighed less for their length, had reduced serum leptin concentrations, and also displayed sex-specific disruption in the expression of hypothalamic neuropeptides on P1. These effects were not associated with maternal changes in circulating metabolic hormones and were therefore unlikely to be due to the altered placental transfer of nutrients. Specifically, the brain fatty acid composition profile in pups indicated a pronounced increase in ω3 PUFAs and a decrease in ω6 PUFAs in those born to ω3-fed dams, reflecting the dietary intake of the mother.

The detrimental effects of poor maternal diet and maternal diet-induced obesity on offspring development are well established [46,47,48], as is the role that maternal diet plays in disrupting the maturation of hypothalamic satiety signalling [13]. The poor neurodevelopmental consequences of maternal obesity have been attributed in part to insufficient intake of ω3 fatty acids [23]. We therefore anticipated that long-term maternal HFSD consumption would lead to poor growth and metabolic outcomes at birth, and that these would be ameliorated by the intake of an ω3-replete diet during pregnancy. Surprisingly, the effects of maternal HFSD on newborn pups were minimal, while ω3 supplementation during pregnancy, particularly when the dam consumed a control chow diet pre-conception, significantly impacted some core developmental indices. Overall, these data demonstrate a substantial impact of maternal ω3 consumption on neuroendocrine parameters in newborn offspring. While long-term effects of these changes remain to be investigated, these findings suggest that a balanced intake of ω3/ω6 PUFAs, rather than a particularly rich ω3 diet, may be essential for healthy development.

Leptin is key for foetal growth and development, including the maturation of the brain [49]. During the first two weeks of life in rodents, leptin facilitates the development and maturation of the hypothalamic satiety-regulatory circuitry [13,50], before acquiring its adult physiological role as a regulator of food intake and energy expenditure [51]. Perinatal changes in leptin levels are associated with an increased risk of cardiovascular and metabolic diseases in adult life [49]. Importantly, both hypo- and hyperleptinemia during the early postnatal period is associated with increased risk to develop obesity later in life [15,21,52,53]. Foetal leptin is derived from multiple sources, including the maternal circulation via transplacental transfer, and leptin synthesis by foetal adipocytes, with its increasing contribution in late gestation [54]. While the placenta itself is able to synthesise leptin, its contribution to foetal leptin is negligible [55,56]. Foetal leptin levels increase near term, followed by a robust decline at birth, indicating that the high leptin levels in the near-term foetus originate primarily from the mother [57]. Postnatal leptin levels are then closely correlated with the concentration of leptin in the maternal milk, at least during the first half of the lactation period [58]. In our study, pups had access to the mother’s milk for only one day. Thus, leptin from maternal milk is unlikely to markedly affect their circulating leptin levels at this timepoint. Our data indicate that maternal intake of an ω3 diet in those fed chow pre-conception led to a significant reduction in leptin levels in newborn offspring on P1. At least in males, this reduction was similar to those born to dams fed HFSD before and during pregnancy, while a switch to a chow or ω3 diet during pregnancy from HFSD pre-conception eliminated this decline in male offspring. Together with reduced body weight for their length, a period of hypo-leptinemia during early development may exacerbate catch-up growth, leading to increased risk of metabolic disease later in life [52]. While reduced growth and leptin levels in rat offspring born to HFSD-fed dams have been previously documented, including in P1 pups [18], similar outcomes in ω3 offspring at birth were unexpected. However, increased dietary intake of ω3 PUFAs during late gestation and lactation has previously been shown to reduce body weight and circulating leptin of P7 pups, compared to those born to dams that consumed a balanced ω6/ω3 diet [59]. Maternal dietary supplementation of ω6 PUFAs has similarly led to reduced pup body weight, and both ω3 and ω6 PUFA intake have been shown to restrict adipocyte size and fat pad weight, relative to pups born to dams fed a balanced ω6/ω3 diet [59]. These changes were attributed to decreased endogenous offspring leptin production [59], a possibility that was not tested with our study design.

It is worth noting that early restricted postnatal growth does not necessarily result in adverse metabolic programming later in life [60]. As such, postnatal nutrient restriction in growth-restricted offspring prevents excess catch-up growth and normalises body weight and leptin levels in the long term [60]. We have also previously shown recovery of hypothalamic leptin resistance and normalisation of hypothalamic satiety pathways after a period of postnatal obesity that led to disruption of the hypothalamic metabolic circuitry [21]. Typically, the development of leptin resistance in adults is mediated by impaired access of leptin to the hypothalamus, with elevated triglycerides inhibiting leptin transport across the blood-brain barrier and disrupting leptin receptor function [61]. Appropriate access of leptin to the brain is essential during the critical period of leptin-induced maturation of hypothalamic feeding circuitry [15]. In the present study, we saw a reduction in pup serum triglycerides in all dietary groups relative to chow-only-fed controls, while no differences were seen in the hypothalamic expression of the leptin receptor between the groups. The effects of triglycerides on leptin transport across the blood-brain barrier have only been established in the adult brain [61]. However, the data presented here suggest that there were no apparent disruptions at this early neonatal phase, ahead of the postnatal leptin surge, that would prevent access of already diminished levels of leptin to the brain. It is important to note that while the blood-brain barrier is functionally effective at birth [62,63], some of the transport mechanisms are different in the developing brain, rendering it more susceptible to disruption [64]. As such, the permeability of the blood-brain barrier in the ARC is increased in the offspring of obese dams [65]. Whether maternal consumption of HFSD or ω3 PUFAs in our study compromises the development of the blood-brain barrier should be the subject of future investigation.

We also assessed expression of some key hypothalamic neuropeptides known to regulate appetite and satiety in the adult brain [66]. While the maturation of neuronal projections between hypothalamic subregions related to feeding and metabolism is not fully attained until the end of the third postnatal week in rodents [14], nearly all of the components of this neuronal circuitry are present in the hypothalamus at birth [44]. Apart from changes in ARC-NPY density, there were no other effects of maternal diet on the expression of metabolic neuropeptides and receptors in the hypothalamus of male pups. In females, however, hypothalamic gene expression of *Agrp* and *Pomc* was increased in pups born to ω3-fed dams. During the first two weeks of postnatal life, only 10–20% of POMC neurons express the leptin receptor compared to 40–50% of NPY/AgRP neurons in the ARC [67]. This colocalization of *Lepr* rises to nearly 80% in both populations of neurons by the end of the third postnatal week, suggesting that leptin predominantly acts on NPY/AgRP and not POMC neurons during the early postnatal period [67]. NPY/AgRP and POMC neurons express free fatty acid receptors [68] that are directly activated by ω3 PUFAs, at least in the adult brain [69]. Together, our results suggest that increased hypothalamic expression of *Agrp* and *Pomc* in ω3 female pups may be driven directly by ω3 PUFAs and leptin-independent signalling. We have previously shown a distinct sex-dependent vulnerability of the developing hypothalamus to neonatal over-nutrition, with males, but not females, exhibiting disrupted hypothalamic satiety signalling during the second week of postnatal life if overfed from birth [20,21,34]. Our data strongly suggest that prenatal ω3 supplementation may also differentially program hypothalamic metabolic signalling pathways in male and female offspring.

The benefits versus risks of a high ω3 PUFA diet are still debated, and maternal intake of a diet either deficient or with excess ω3 PUFAs appears capable of disrupting neurodevelopment and metabolic status later in life [70,71,72]. In our study, the ratio of ω3/ω6 PUFAs was 7:1 in the ω3-replete diet; 1:9 in HFSD and 1:3.5 in the chow-control fed. Previous work has shown maternal dietary intake of a significantly higher ω3/ω6 ratio of 14:1 throughout pregnancy and lactation resulted in adverse postnatal and adult outcomes in the offspring [70,72]. While a direct comparison between the composition of the diet used in our study to ω3 PUFA amounts in human studies is not possible due to the different types of ω3 PUFAs, differences in doses and timing of supplementation [73,74], the ω3 diet used here was nearly identical to that previously shown to suppress placental oxidative stress and to enhance placental and foetal growth in Wistar rats [31], with both the diet and the animals sourced from the same suppliers as in our study. Consumption of this same diet during pregnancy has also been shown to enhance the capacity of the placenta to resolve inflammation, by elevating the placental expression of potent anti-inflammatory and pro-resolving lipid mediators [75]. Maternal intake of this ω3-high diet has also been shown to directly influence placental and foetal fatty acid composition, with an increase in ω3 and decrease in ω6 PUFAs in both maternal and foetal tissues [31]. Similarly, our findings show a robust increase in ω3 and a decrease in ω6 fatty acids in the brains of pups born to ω3-fed dams, irrespective of consumption of either chow or HFSD pre-conception. These findings clearly indicate normal transplacental transport of PUFAs. It is important to note that due to the mating protocol utilised in our study, we were unable to assess exact gestation length. However, a reduced body weight in newborn ω3 pups is unlikely to indicate premature birth. ω3 PUFAs typically increase gestation length due to their potent anti-inflammatory actions [76]. We also see reductions in body weight relative to length in our pups, suggesting that weight is affected independently of overall growth. Noteworthy, we have used a standard laboratory chow feed as a control diet in this study. Although we and others have previously incorporated a standard laboratory feed in studies with a nutritional focus [18,53,77,78,79,80], and while the composition of the diets in this study had a matched protein content of 19.4–20%, the use of unrefined grain-based diets compared to diets made with refined, purified ingredients may affect this comparison [81]. It would therefore be of interest for future enquiries to compare the effects of HFSD and high-ω3 to a control purified diet in the context of perinatal development.

In conclusion, our current results suggest that, in male pups, gestational intake of a diet rich in ω3 PUFAs may alter rBMI and circulating leptin levels in a similar way to maternal HFSD consumption, typically associated with adverse metabolic programming. While the long-term implications of these changes are currently unknown, these data indicate the importance of a balanced nutritional intake in pregnancy and highlight the need for further animal and clinical studies examining the impact of maternal ω3 intake on offspring development and long-term health. The latter is particularly important given the conflicting clinical evidence on benefits versus risks associated with high ω3 intake, increasing the likelihood of prolonged gestation [82]. Although a direct comparison between the composition of an ω3-replete diet used in the current study and human ω3 intake is not possible, our findings indicate that excessive ω3 consumption during pregnancy resulting in high ω3:ω6 dietary ratio may be detrimental for metabolic development of the offspring.

## Figures and Tables

**Table 1:** Nutritional parameters and fatty acids composition of chow, HFSD and ω3 diets.

Calculated Nutritional Parameters	Diet		
	Chow	HFSD	ω3
Protein (%)	20.0	19.4	19.4
Total Fat (%)	4.8	20.0	5.0
Total Carbohydrate (%)	59.40	No data	63.3
Sucrose (%)	0.0	39.60	10.0
Digestible Energy (MJ/kg)	14.0	18.4	15.8
Total Calculated Energy From Protein (%)	23.0	19.0	10.0
Total Calculated Energy From Lipids (%)	12.0	36.0	22.0
**Calculated Fatty Acid Composition (%)**			
Linoleic Acid 18:2 ω6	1.30	2.90	0.06
α-Linolenic Acid 18:3 ω3	0.30	0.30	0.02
Arachidonic Acid 20:4 ω6	0.01	No data	0.08
EPA 20:5 ω3	0.02	No data	0.27
DHA 22:6 ω3	0.05	No data	1.19
Total ω3	0.37	0.31	1.66
Total ω6	1.31	2.90	0.23
Total Monounsaturated Fats	2.00	7.32	1.10
Total Polyunsaturated Fats	1.77	3.32	1.91
Total Saturated Fats	0.74	9.30	1.67

HFSD: high-fat-high-sugar diet. Data are from Specialty Feeds, Glen Forrest, WA, Australia.

**Table 2:** Primer details.

Target Gene	NCBI Reference Sequence	Taqman Assay ID	Product Size
*Gapdh*	NM_017008.3	4352338E	63
*Lepr*	NM_012596	Rn01433205_m1	94
*Npy*	NM_012614.2	Rn00561681_m1	63
*Agrp*	NM_033650.1	Rn01431703_g1	67
*Pomc*	NM_139326.2	Rn00595020_m1	92
*Ghsr*	NM_032075.3	Rn00821417_m1	61

**Table 3:** Maternal metabolic hormones.

Pre-Conception Diet	Chow		HFSD		
Post-Conception Diet	Chow	ω-3	HFSD	Chow	ω-3
Leptin (ng/mL)	28.4 ± 3.9	48.0 ± 4.2	46.6 ± 6.1	36.8 ± 5.9	69.8 ± 10.7 *^,$^
Triglycerides (mg/dL)	47.2 ± 5.3 ^#^	42.28 ± 3.6 ^#^	105.4 ± 12.3	41.8 ± 3.9 ^#^	46.6 ± 4.1 ^#^
Insulin (ng/mL)	3.3 ± 0.5	4.9 ± 0.8	4.7 ± 1.2	3.0 ± 0.4	4.3 ± 0.5
Total ghrelin (pg/mL)	2172.6 ± 272.6	1729.1 ± 197.5	2531.9 ± 311.1	2193.9 ± 197.5	1885.9 ± 168.9
Acyl ghrelin (pg/mL)	710.5 ± 114.2	769.6 ± 134.9	505.8 ± 81.1	638.5 ± 126.7	563.9 ± 87.4
Des-acyl ghrelin (pg/mL)	1462 ± 246.0	959.6 ± 119.9 ^#^	2026.0 ± 306.4	1387.0 ± 325.4	1247.0 ± 55.9

HFSD: high-fat-high-sugar diet. *Post hoc* differences: * versus the chow-chow group; ^#^ versus the HFSD-HFSD group; ^$^ versus the HFSD-chow group. *p* < 0.05. Data are mean ± SEM (*n* = 6–10).

## Data Availability

Data are available upon reasonable request.

## Supplementary Materials

The following are available online at https://www.mdpi.com/2072-6643/13/1/89/s1, Table S1: Brain fatty acid composition (% total fatty acids; male offspring), Table S2: Brain fatty acid composition (% total fatty acids; female offspring).
