# Erratum: Xavier et al. High Maternal Omega-3 Supplementation Dysregulates Body Weight and Leptin in Newborn Male and Female Rats: Implications for Hypothalamic Developmental Programming. *Nutrients* 2021, *13*, 89

**DOI:** 10.3390/nu13072418

**Published:** 2021-07-15

**Authors:** Soniya Xavier, Jasmine Gili, Peter McGowan, Simin Younesi, Paul F. A. Wright, David W. Walker, Sarah J. Spencer, Luba Sominsky

**Affiliations:** 1 School of Health and Biomedical Sciences, RMIT University, Melbourne, VIC 3083, Australia; soniya.xavier@student.rmit.edu.au (S.X.); jasminegili97@hotmail.com (J.G.); Simin.Younesi@student.rmit.edu.au (S.Y.); paul.wright@rmit.edu.au (P.F.A.W.); David.Walker@rmit.edu.au (D.W.W.); Luba.Sominsky@rmit.edu.au (L.S.); 2 School of Science, RMIT University, Melbourne, VIC 3001, Australia; peter.mcgowan@rmit.edu.au; 3 ARC Centre of Excellence for Nanoscale Biophotonics, RMIT University, Melbourne, VIC 3001, Australia

The authors would like to correct a typographical error in their recently published paper [1]. In the original article, there was a mistake in Table 1 as published. In Table 1, the correct Total Calculated Energy From Protein for ω3 diet is 22% (instead of 10%) and Total Calculated Energy From Lipids for ω3 diet is 10% (instead of 22%). The corrected Table 1 appears below. The authors apologize for any inconvenience caused and state that the scientific conclusions are unaffected. The original article has been updated.

## Figures and Tables

**Table 1:** Nutritional parameters and fatty acids composition of chow, HFSD and ω3 diets.

Calculated Nutritional Parameters	Diet		
	Chow	HFSD	ω3
Protein (%)	20.0	19.4	19.4
Total Fat (%)	4.8	20.0	5.0
Total Carbohydrate (%)	59.40	No data	63.3
Sucrose (%)	0.0	39.60	10.0
Digestible Energy (MJ/kg)	14.0	18.4	15.8
Total Calculated Energy From Protein (%)	23.0	19.0	22.0
Total Calculated Energy From Lipids (%)	12.0	36.0	10.0
**Calculated Fatty Acid Composition (%)**			
Linoleic Acid 18:2 ω6	1.30	2.90	0.06
α-Linolenic Acid 18:3 ω3	0.30	0.30	0.02
Arachidonic Acid 20:4 ω6	0.01	No data	0.08
EPA 20:5 ω3	0.02	No data	0.27
DHA 22:6 ω3	0.05	No data	1.19
Total ω3	0.37	0.31	1.66
Total ω6	1.31	2.90	0.23
Total Monounsaturated Fats	2.00	7.32	1.10
Total Polyunsaturated Fats	1.77	3.32	1.91
Total Saturated Fats	0.74	9.30	1.67

HFSD: high-fat-high-sugar diet. Data are from Specialty Feeds, Glen Forrest, WA, Australia.
