# Supplementary material for: High Maternal Omega-3 Supplementation Dysregulates Body Weight and Leptin in Newborn Male and Female Rats: Implications for Hypothalamic Developmental Programming
Source: Nutrients. 2020 Dec 30;13(1):89. doi: 10.3390/nu13010089 (PMC7823471; doi:10.3390/nu13010089)
Supplement: Supplementary file 1 [file nutrients-13-00089-s001.pdf]

Supplementary Table 1. Brain fatty acid composition (% total fatty acids; male offspring).

Pre-conception diet	**Chow**		**HFSD**		
Post-conception diet	**Chow**	**ω3**	**HFSD**	**Chow**	**ω3**
C16:0	33.1 ± 0.4	29.9 ± 0.8 [*]	32.0 ± 0.7	31.3 ± 0.2	30.3 ± 1.0 [*]
C16:1ω-7	2.1 ± 0.1	2.3 ± 0.1	2.2 ± 0.1	2.1 ± 0.1	2.1 ± 0.1
C18:0	16.9 ± 0.3	16.4 ± 0.6	16.4 ± 0.5	16.6 ± 0.1	15.5 ± 0.7
C18:1ω-9	12.6 ± 0.3	13.1 ± 0.4	12.6 ± 0.3	12.6 ± 0.2	13.2 ± 0.5
C18:2ω-6	0.9 ± 0.0	0.6 ± 0.0 [*, $]	0.6 ± 0.0 [*, $]	0.9 ± 0.0	0.5 ± 0.1 [*, $]
C20:1ω-9	0.2 ± 0.0	0.2 ± 0.0	0.2 ± 0.0	0.2 ± 0.0	0.2 ± 0.1
C20:2ω-6	0.2 ± 0.0	0.0 ± 0.0	0.1 ± 0.1	0.1 ± 0.1	0.0 ± 0.0
C20:3ω-6	0.5 ± 0.0	0.4 ± 0.0	0.4 ± 0.0	0.5 ± 0.0	0.4 ± 0.0
C20:4ω-6	12.9 ± 0.3	9.3 ± 0.2 [*]	14.0 ± 0.6 [^, “]	14.0 ± 0.2 [^, “]	9.4 ± 0.6 [*]
C20:5ω-3	0.2 ± 0.1	1.1 ± 0.1 [*]	0.1 ± 0.1 [^, “]	0.2 ± 0.1 [^, “]	1.2 ± 0.2 [*]
C22:1ω-9	0.2 ± 0.1	0.1 ± 0.1	0.2 ± 0.1	0.1 ± 0.1	0.1 ± 0.1
C22:6ω-3	13.5 ± 0.6	20.0 ± 1.3 [*]	13.8 ± 1.1 [^, “]	14.8 ± 0.4 [^, “]	19.2 ± 1.2 [*]
Total PUFAs	43.4 ± 0.7	47.4 ± 1.2	44.5 ± 1.4	45.7 ± 0.5	46.6 ± 1.4
Total ω-3	13.7 ± 0.6	21.2 ± 1.3 [*]	13.9 ± 1.1 [^, “]	15.0 ± 0.4 [^, “]	20.3 ± 1.3 [*]
Total ω-6	14.8 ± 0.4	10.6 ± 0.3 [*]	15.0 ± 0.6 [^, “]	15.8 ± 0.2 [^, “]	10.6 ± 0.6 [*]
Total ω-9	12.9 ± 0.3	13.4 ± 0.5	12.9 ± 0.4	12.9 ± 0.2	13.6 ± 0.6

HFSD: high-fat-high-sugar diet. PUFAs: polyunsaturated fatty acids. Data were analysed by two-way ANOVA followed by Tukey's *post hoc* tests. *Post hoc* differences: * versus the chow-chow group; ^ versus the chow-ω3 group; # versus the HFSD-HFSD group; $ versus the HFSD-chow group; “ versus the HFSD-ω3 group. $p < 0.05$. Data are mean ± SEM (n = 4-5 per group).

Supplementary Table 2. Brain fatty acid composition (% total fatty acids; female offspring).

Pre-conception diet	Chow		HFSD		
Post-conception diet	**Chow**	**ω3**	**HFSD**	**Chow**	**ω3**
C16:0	35.9 ± 1.9	33.2 ± 1.0	35.6 ± 1.7	38.2 ± 3.3	34.1 ± 0.2
C16:1ω-7	1.8 ± 0.1	1.9 ± 0.1	2.1 ± 0.2	1.7 ± 0.2	2.1 ± 0.0
C18:0	20.4 ± 1.7	19.3 ± 0.9	20.0 ± 1.6	21.9 ± 2.2	19.4 ± 0.3
C18:1ω-9	13.7 ± 0.7	13.9 ± 0.4	13.8 ± 0.7	11.7 ± 2.2	14.0 ± 0.3
C18:2ω-6	0.8 ± 0.1	0.6 ± 0.0	0.6 ± 0.1	0.7 ± 0.1	0.5 ± 0.0 *
C20:1ω-9	0.2 ± 0.0	0.2 ± 0.0	0.2 ± 0.0	0.1 ± 0.1	0.2 ± 0.0
C20:2ω-6	0.2 ± 0.0	0.2 ± 0.0	0.2 ± 0.0	0.2 ± 0.1	0.2 ± 0.0
C20:3ω-6	0.4 ± 0.1	0.4 ± 0.1	0.4 ± 0.0	0.3 ± 0.2	0.4 ± 0.0
C20:4ω-6	10.0 ± 2.0	7.4 ± 0.6	10.7 ± 1.6	8.6 ± 2.3	7.3 ± 0.4
C20:5ω-3	0.1 ± 0.1	0.9 ± 0.1 *	0.1 ± 0.0 ^, “	0.1 ± 0.1 ^, “	0.8 ± 0.0 *
C22:1ω-9	0.3 ± 0.1	0.2 ± 0.0	0.2 ± 0.0	0.3 ± 0.1	0.3 ± 0.0
C22:6ω-3	9.7 ± 2.6	15.0 ± 1.7	9.2 ± 2.2	7.9 ± 2.4	13.6 ± 0.0
Total PUFAs	37.9 ± 4.0	41.4 ± 2.0	38.1 ± 3.4	32.3 ± 7.1	40.0 ± 0.5
Total ω-3 m	10.0 ± 2.6	16.1 ± 1.7	9.4 ± 2.2	8.1 ± 2.5	14.5 ± 0.5
Total ω-6	11.9 ± 2.1	9.0 ± 0.6	12.3 ± 1.7	10.3 ± 2.7	8.8 ± 0.4
Total ω-9	14.3 ± 0.8	14.4 ± 0.3	14.3 ± 0.6	12.2 ± 1.9	14.7 ± 0.4

HFSD: high-fat-high-sugar diet. PUFAs: polyunsaturated fatty acids. Data were analysed by two-way ANOVA followed by Tukey's *post hoc* tests. *Post hoc* differences: * versus the chow-chow group; ^ versus the chow-ω3 group; # versus the HFSD-HFSD group; $ versus the HFSD-chow group; “ versus the HFSD-ω3 group. m – main effect of pregnancy diet (with no *post hoc* differences). $p < 0.05$. Data are mean ± SEM (n = 3-5 per group).
